# Clinical detection of corticobasal syndrome, beyond an “atypical" Alzheimer's

**DOI:** 10.1002/alz70857_102408

**Published:** 2025-12-24

**Authors:** Ignacio Flores, Carmela Tartaglia, Gabor G. Kovacs, Blas Couto

**Affiliations:** ^1^ Neuroscience Institute, Favaloro Foundation, Buenos Aires, Buenos Aires, Argentina; ^2^ Instituto de neurociencias Fundacion Favaloro, Buenos Aires, buenos aires, Argentina; ^3^ Rossy PSP Program, University Health Network and the University of Toronto, Toronto, ON, Canada; ^4^ Division of Neurology, Toronto Western Hospital, University Health Network Memory Clinic, Toronto, ON, Canada; ^5^ Tanz Centre for Research in Neurodegenerative Diseases, University of Toronto, Toronto, ON, Canada; ^6^ Laboratory of Progressive Neuromotor and Neurobehavioral Disorders at Instituto de Neurociencia Cognitiva y Traslacional (INCYT), CABA, Buenos Aires, Argentina; ^7^ Unidad de Movimientos Anormales, Institute of Neuroscience of the Fundacion Favaloro, CABA, Buenos Aires, Argentina

## Abstract

Corticobasal syndrome (CBS) corresponds to a progressive deterioration of motor and associative cognitive functions that include ideomotor apraxia, astereognosis/loss of graphesthesia, sensory extinction, alien limb in combination with marked asymmetric limb dystonia, rigidity or myoclonus. CBS can be the initial manifestation of early‐onset atypical Alzheimer's Disease (AD). Although early memory impairment has been proposed as a proxy to AD etiology of CBS, several CBS caused by corticobasal degeneration or progressive supranuclear palsy neuropathological change, do not ever develop a progressive memory impairment to the point of reaching moderate‐severe dementia. In this session, we will stress the details in history taking and findings on physical examination as well as neurocognitive profile that early in the disease course help distinguish those patients with CBS of potential AD cause (CBS‐ADNP).

